# Supplementary material for: Monitoring Therapeutic Responses to Silicified Cancer Cell Immunotherapy Using PET/MRI in a Mouse Model of Disseminated Ovarian Cancer
Source: Int J Mol Sci. 2022 Sep 10;23(18):10525. doi: 10.3390/ijms231810525 (PMC9504323; doi:10.3390/ijms231810525)

Supplemental Data

Monitoring therapeutic responses to silicified cancer cell immunotherapy using PET/MRI in a mouse model of disseminated ovarian cancer

Erik N. Taylor, Colin M. Wilson*, Stefan Franco, Henning De May, Lorél Y. Medina Yirong Yang, Erica B. Flores, Eric Bartee, Reed G. Selwyn, Rita E. Serda*

C.M. Wilson, E.N. Taylor, R.G. Selwyn. Department of Radiology, University of New Mexico Health Science Center, Albuquerque, NM 87131, USA; S. Franco, L.Y. Medina, E.B. Flores, E. Bartee, R.E. Serda. Internal Medicine, University of New Mexico Health Science Center, Albuquerque, NM 87131, USA; H. De May, Department of Obstetrics & Gynecology, University of New Mexico Health Science Center, Albuquerque, NM 87131, USA; Y. Yang, Pharmaceutical Sciences, University of New Mexico Health Science Center, Albuquerque, NM 87131, USA

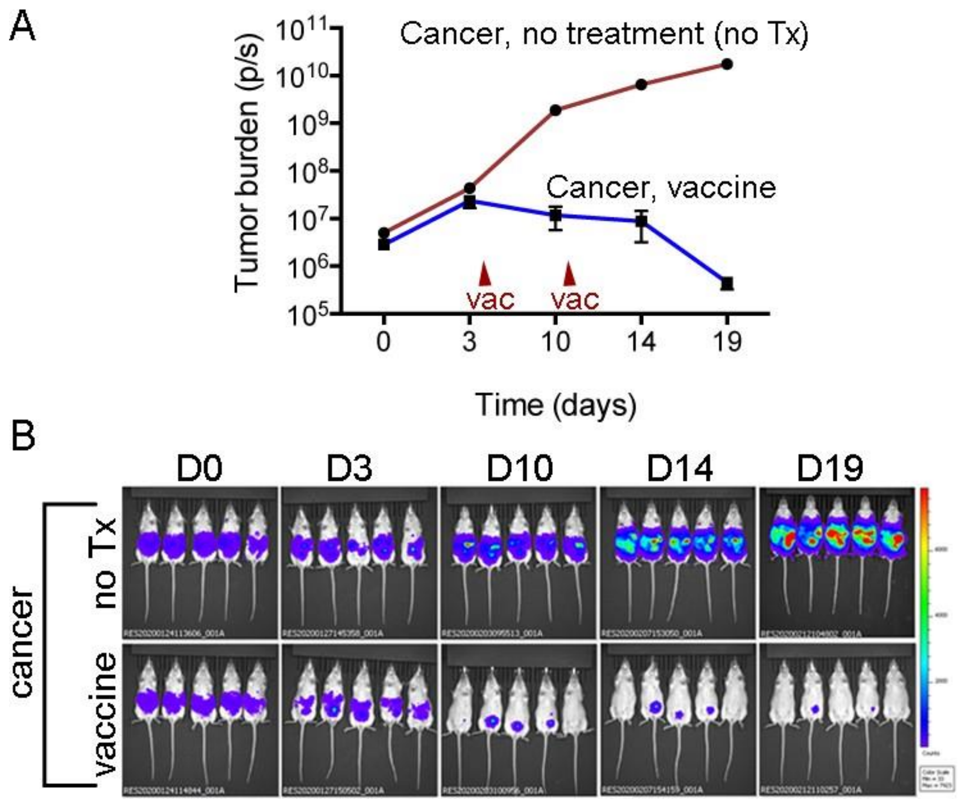

Figure S1. Additional efficacy data on therapeutic vaccination of mice with disseminated ovarian cancer. Bioluminescent images (**A**) and graphs (**B**) of tumor burden (photons/s) for FVB mice injected IP with 2x10^{5} BR5-*akt*-Luc2 cells (Day 0) and vaccinated with 3x10^{6} BR5-*akt* vaccine cells (Days 4 and 11).

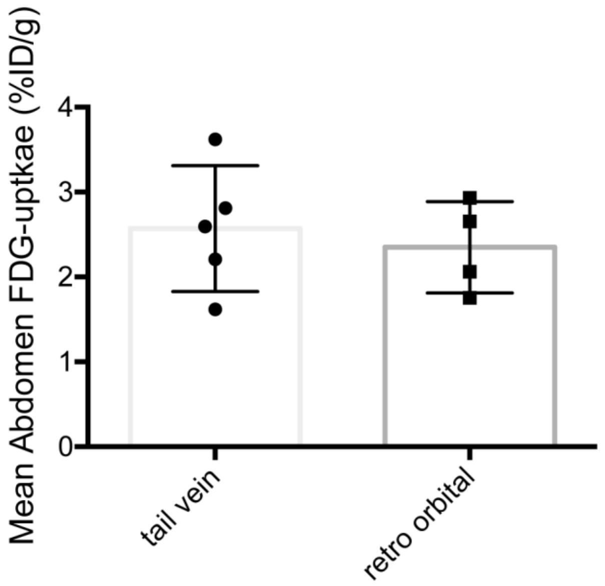

Figure S2. Impact of injection route on abdominal FDG uptake. Abdominal FDG-uptake (%ID/g) in untreaated, tumor-bearing mice following either retro-orbital (n=4) or tail vein (n=5) injection of [^{18}F]FDG. Statistical analysis was performed using an unpaired, parametric, two-tailed t-test. No significant differences were observed in abdominal uptake between the different injection methods.

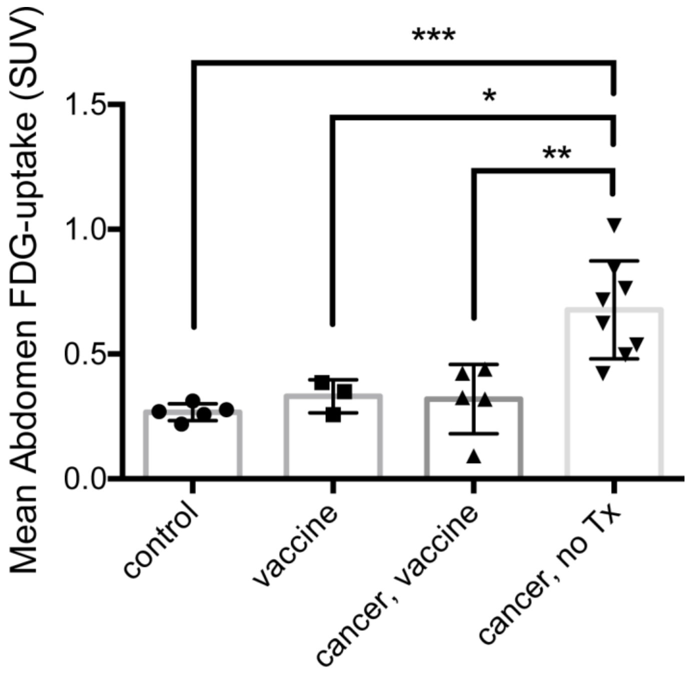

Figure S3. Abdominal FDG-uptake using the Standardized Uptake Value (SUV). Abdominal [18F]FDG concentration normalized by injected dose per unity body weight in control (naïve), vaccinated (+/- cancer), and untreated cancer bearing mice. Statistical analysis between groups was carried out using ANOVA with multiple comparisons by Tukey's test. The comparisons were significantly different as indicated by * $p \leq 0.05$, ** $p \leq 0.01$, and ***$p \leq 0.001$.

Figure S4. Whole blood glucose levels in mice pre-[18F]FDG administration. Pre-scan whole blood glucose (PreWBglc) levels were similar treatment groups.

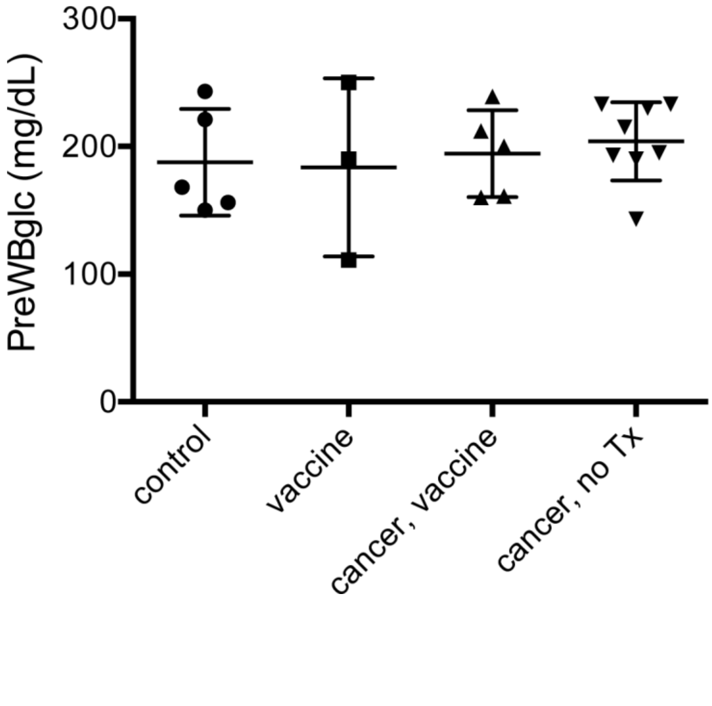

Supplement: Supplementary file 1 [file ijms-23-10525-s001.zip › ijms-1858590-supplementary.pdf]
